# Effects of Berberine on the Gastrointestinal Microbiota

**DOI:** 10.3389/fcimb.2020.588517

**Published:** 2021-02-19

**Authors:** Lichao Zhang, Xiaoying Wu, Ruibing Yang, Fang Chen, Yao Liao, Zifeng Zhu, Zhongdao Wu, Xi Sun, Lifu Wang

**Affiliations:** ^1^ Department of Parasitology of Zhongshan School of Medicine, Sun Yat-sen University, Guangzhou, China; ^2^ Key Laboratory of Tropical Disease Control, Ministry of Education, Sun Yat-sen University, Guangzhou, China; ^3^ Provincial Engineering Technology Research Center for Biological Vector Control, Guangzhou, China; ^4^ Department of Gastroenterology, Third Affiliated Hospital of Sun Yat-sen University, Guangzhou, China; ^5^ Medical Department, Xizang Minzu University, Xianyang, China; ^6^ School of Medicine, South China University of Technology, Guangzhou, China

**Keywords:** berberine, intestinal flora, obesity, diabetes, hyperlipidemia

## Abstract

The gastrointestinal microbiota is a multi-faceted system that is unraveling novel contributors to the development and progression of several diseases. Berberine has been used to treat obesity, diabetes mellitus, atherosclerosis, and metabolic diseases in China. There are also clinical trials regarding berberine use in cardiovascular, gastrointestinal, and endocrine diseases. Berberine elicits clinical benefits at standard doses and has low toxicity. The mechanism underlying the role of berberine in lipid‐lowering and insulin resistance is incompletely understood, but one of the possible mechanisms is related to its effect on the gastrointestinal microbiota. An extensive search in electronic databases (PubMed, Scopus, Embase, Web of Sciences, Science Direct) was used to identify the role of the gastrointestinal microbiota in the berberine treatment. The aim of this review was to summarize the pharmacologic effects of berberine on animals and humans by regulation of the gastrointestinal microbiota.

## Introduction

Berberine is a quaternary ammonium salt from the protoberberine group of isoquinoline alkaloids(Caliceti et al., 2016). The latter are present in the roots, bark, and other structures of plants found typically in traditional Chinese/East Asian medicines. Such plants include *Coptis chinensis*, *Berberis aristata*, *B. petiolaris*, *B. vulgaris*, *B. aquifolium*, and *B. thunbergii* (Cicero and Baggioni, 2016). Unusually high berberine content has been reported in *Phellodendron amurense* and *C. chinensis* (Habtemariam, 2016).

Many people in China use traditional herbal formulations to treat diseases, as recorded in the *Pharmacopoeia of China* (2015). Such formulations have excellent efficacy, including clearing away heat, resolving dampness, purging fire, and detoxification (Wang et al., 2017a). These formulations have been used to treat pharyngolaryngitis, typhoid, gastroenteritis, diabetes mellitus (DM), and secretory diarrhea for more than 1,000 years in China (Yang et al., 2010).

Berberine and its derivatives display several pharmacologic effects through various mechanisms (Jin et al., 2016). Berberine may be therapeutic against various types of chronic diseases, such as obesity, DM, inflammatory bowel disease (IBD), atherosclerosis, Alzheimer’s disease, rheumatoid arthritis, and cardiovascular diseases, due to its multiple-target effects (Jin et al., 2016). Also, the binding of berberine with histone–DNA complexes can cause interferences in vital cellular processes, such as cell division and cause the death of cancer cells by activating the apoptosis in living cells (Chen et al., 2016; Hasanein et al., 2017; Roudini et al., 2019). In vitro, berberine has important anti-inflammatory and antioxidant activities.

In animal models, berberine has neuro-protective and cardiovascular-protective effects. Tan et al. showed that berberine was distributed rapidly (in a descending order of its amount) in the brain, lungs, heart, liver, kidneys, muscle, pancreas, and fat in rats (Tan et al., 2013). The mechanism of action of berberine is associated with its regulatory effect on cellular targets, such as the low-density lipoprotein receptor (LDLR), insulin receptor (IR), adenosine monophosphate-activated protein kinase(AMPK), proprotein convertase subtilisin kexin-9, protein tyrosine phosphatase-1B(PTP-1B), mitochondrial adenosine triphosphate (ATP) production, and brown fat tissue (Kong et al., 2004).

## The Gastrointestinal Microbiota (GM)

The GM (also referred to as the “gut flora” or “gut microbiota”) are the microorganisms that live in the digestive tracts of mammals, including bacteria, archaea, viruses, fungi, and some parasites. Most of the GM reside in the distal large intestine. Dysbiosis refers to an alteration in the quality and/or quantity of the GM; such changes can influence the physiology of the host and lead to the onset of various diseases (Kuno et al., 2016).

Research carried out over the last few years indicates that the GM represents an important factor for the regulation of body health and may be closely related to the pathogenesis of obesity (Walker and Parkhill, 2013), diabetes mellitus (DM), inflammation (Kamada et al., 2013), cardiovascular diseases and cancer (Louis et al., 2014), and other diseases (Round and Mazmanian, 2009). The GM is composed of several phyla, including *Bacteroidetes*, *Firmicutes*, *Proteobacteria*, *Verrucomicrobia*, *Actinobacteria*, *Fusobacteria*, and *Cyanobacteria* et al (Sekirov et al., 2010). *Bacteroidetes* and *Firmicutes* are known to represent the main components of the GM. An imbalance in the ratio of *Firmicutes* and *Bacteroidetes* (F:B) in the GM has been associated with several diseases (Gao et al., 2017). Recent evidence also suggests that the GM plays a role in homeostasis and may exert positive influence on immune responses and prevent the development of inflammatory diseases. Berberine has been shown to affect the bacteria that produce short-chain fatty acids (SCFAs) in the GM (Wang et al., 2017b). Research has also shown that SCFA-producing bacteria benefit the host by protecting the mucosa from damage induced by pathogens, by providing colonocyte nutrients, and by mitigating inflammation (Maslowski et al., 2009).

## The Effects of Berberine on the GM

The GM is also known to affect drug metabolism, both directly and indirectly, and particularly with regards to drugs that are administered orally. Berberine reduces the levels of lipids and glucose in the blood *via* multi-target mechanisms, including modulation of the GM composition (Zhang et al., 2012). Berberine is also known to reduce the diversity of the GM and interfere with the relative abundance of *Desulfovibrio*, *Eubacterium*, and *Bacteroides* (Cui et al., 2018b). In addition, *Bacteroides* were shown to be enriched in the colon and terminal ileum of mice (C57BL/6) treated with berberine, but berberine treatment reduced the populations of *Ruminococcus gnavu* (Genus of *Mediterraneibacter*)*, Ruminococcus schinkii* (Genus of *Blautia*), *Lactobacillus acidophilus* (Genus of *Lactobacillus*)*, Lactobacillus murinus* (Genus of *Ligilactobacillus*), and *Lactococcus lactis* (Genus of *Lactococcus*) (Guo et al., 2016). Recent studies have shown that berberine has beneficial effects on the immune cells of the intestinal immune system and affects the expression of several intestinal immune factors. Berberine has also been shown to inhibit the mRNA expression of *interleukin (IL)-1β*, *IL-4*, *IL-10*, *macrophage migration inhibitory factor (MIF*), and *tumor necrosis factor (TNF)-α*, while also reducing low-grade inflammation (Gong et al., 2017). Short-term exposure to berberine alters the populations of intestinal bacteria by reducing the activity of *Clostridium* cluster XIVa and IV, and their bile salt hydrolase (BSH), thus leading to the accumulation of taurocholic acid (TCA). TCA can activate intestinal farnesoid X receptor (FXR) which can then mediate the metabolism of bile acids, lipids, and glucose (Tian et al., 2019). Butyrate is a short-chain fatty acid (SCFA) produced during fermentation of fibers and other substrates by an anaerobic bacteria resident in the gastrointestinal tract (Roediger et al., 1982). Berberine has also been shown to enrich the population of butyrate-producing bacteria in the GM, thus promoting the synthesis of butyrate *via* the acetyl CoA-butyryl CoA-butyrate pathway. Subsequently, the butyrate enters the blood and reduces the levels of lipids and glucose (Wang et al., 2017b).

The GM is known to play a key role in the development of metabolic disorders. One factor underlying the application of berberine treatment is that berberine can increase the rates of cellular glucose uptake and metabolism (Cok et al., 2011). Other research studies are investigating the effects of berberine against cancer. In this article, we review the role of the GM on non-transmissible diseases following berberine treatment.

## The Effects of Berberine on Obesity

The global obesity epidemic is prompting significant efforts to identify host and environmental factors that affect energy balance in the human body. For example, Turnbaugh et al. reported that host obesity is related to an increase in the intestinal F:B ratio (Turnbaugh et al., 2006). Berberine has been shown to revert the structural changes in the GM induced by a high-fat diet and regulate diversity in the GM. Berberine has also been shown to change 134 operational taxonomic units (OTUs) that were identified by nearest shrunken centroids analysis in obese rats induced by a high-fat diet, and was also associated with changes in obesity phenotypes. Sixty of the 134 OTUs were significantly increased with berberine treatment, particularly those belonging to putative SCFA-producing bacteria, including *Allobaculum*, *Bacteroides*, *Blautia*, *Butyricicoccus*, and *Phascolarctobacterium*.

A previous study showed that there is a reduced abundance of *A. muciniphila* in obese humans (Everard et al., 2013). Interestingly, others discovered that *A. muciniphila* was less abundant in the intestinal microbiota of both genetic and diet-induced obese and diabetic mice (Schneeberger et al., 2015; Leal-Diaz et al., 2016). *Akkermansia* spp. abundance was markedly increased in HFD-fed mice treated with berberine (Zhu et al., 2018). *A. muciniphila* treatment was shown to reverse metabolic disorders induced by high-fat diets, including fat-mass gain, metabolic endotoxemia, adipose tissue inflammation, and insulin resistance (IR). The administration of *A. muciniphila* was also shown to increase the intestinal levels of endocannabinoids that control inflammation, the gut barrier, and the secretion of peptides in the gut (Everard et al., 2013). Increased levels of colonization by *A. muciniphila* were also shown to induce the expression of low-density lipoprotein receptors and apolipoprotein E in the hepatocytes of CREBH-null mice (C57BL/6J). This facilitated the uptake of intermediate-density lipoprotein *via* the mediation of apolipoprotein B100 and apolipoprotein E, thus leading to the increased clearance of triglyceride-rich lipoprotein remnants, chylomicron remnants, and intermediate-density lipoproteins, from general circulation. The oral administration of *A. muciniphila* also improved hepatic endoplasmic reticulum stress and metabolic inflammation in CREBH-null mice (Shen et al., 2016).

In a previous study, Sun et al. reported that berberine improved metabolic disorders caused by a high-fat diet and did so by regulating the GM–gut–brain axis. Berberine also increased the ratio of B:F and the proportion of SCFA-producing bacteria, thus promoting the increased expression of glucagon-like peptide (GLP)-1 in intestinal L cells, a type of endocrine cell in the intestine that secretes GLP-1 (Sun et al., 2016). Other research reported a positive correlation between the abundance of bacteria belonging to the *Akkermansia* genus and the number of L-cells in the colon; the administration of *A. muciniphila* significantly increased GLP-1 release from colonic L-cells (Everard et al., 2013).

Zhang et al. also reported that berberine modulated the GM, enriched the population of SCFA-producing bacteria, and regulated microbial diversity, thus enhancing intestinal integrity (Zhang et al., 2015). These authors also revealed that *Phascolarctobacterium*, *Anaerotruncus*, and *Oscillibacter*, may be solely responsible for the beneficial effects of berberine on intestinal permeability. Berberine increased the expression of *ZO-1* mRNA by inhibiting the abundance of *Oscillibacter*, thus antagonizing obesity.

Berberine can enrich the population of butyrate-producing bacteria in the GM (Wang et al., 2017b). Butyrate-induced upregulation of GLP-1 and PYY may be important in preventing or treating obesity and insulin resistance (Vidrine et al., 2014). Treatments with butyrate or increasing butyrate productions have been shown to prevent or attenuate obesity and insulin resistance (Li M. et al., 2016; Li X. et al., 2016; Goldsmith et al., 2017; Zhang et al., 2017). Butyrate has also been shown to increase B-adrenergic receptor profiles in adipocytes, which occurs *via* HDACi activity, a similar mechanism for upregulating fatty acid oxidation may occur in white adipose tissue (McNabney and Henagan, 2017). The HDACi activity of butyrate has also been associated with its ability to prevent adipose tissue inflammation, a contributing factor to insulin resistance during obesity (Ding et al., 2000; Wang et al., 2015). N-(1-carbamoyl-2-phenyl-ethyl) butyramide (FBA), a synthetic more palatable derivative of butyrate, in mice fed the HFD reduces hepatic fat accumulation and decreases metabolic/mitochondrial efficiency, counteracting obesity, IR, and inflammation (Mollica et al., 2017). Elevation of SCFA availability by increasing dietary fiber intake or diet supplementation with butyrate may prevent the development of metabolic disarrangements and the insulin resistance associated with obesity (Galisteo et al., 2008; Nilsson et al., 2010).

## The Effects of Berberine on Hyperlipidemia

Hyperlipidemia is a major component of the metabolic syndrome, and gives rise to increased levels of triglyceride (TG), total cholesterol (TC), and low-density lipoprotein cholesterol (LDL-C), while reducing the serum levels of high-density lipoprotein cholesterol (HDL-C) (Chen et al., 2014). Berberine has been shown to exert a therapeutic effect on patients with hyperlipidemia (Kong et al., 2004).

The “one-drug-multiple-target” concept is characteristic of the treatment applied for hyperlipidemia. The main targets of berberine on the metabolism of lipids and glucose are IR and the LDLR. Interestingly, berberine has been shown to reduce the serum levels of lipids in humans, hamsters, rats, and mice (Kong et al., 2004; Chang et al., 2010; Wang et al., 2010). A clinical trial showed that berberine treatment reduced the levels of total cholesterol, triglycerides, and LDL cholesterol, while increasing the levels of HDL cholesterol after 3 months of treatment when compared with a placebo (Derosa et al., 2013).

In another study, the oral administration of berberine in 32 hypercholesterolemic patients for 3 months was shown to reduce serum levels of cholesterol by 29%, triglycerides by 35% and LDL-cholesterol by 25% (Kong et al., 2004). Furthermore, the treatment of hyperlipidemic hamsters with berberine reduced serum levels of cholesterol by 40% and LDL-cholesterol by 42%, with a 3.5-fold increase in hepatic *LDLR* mRNA expression and a 2.6-fold increase in hepatic LDLR protein levels (Kong et al., 2004; Yao et al., 2015). Berberine has also been shown to increase the abundance of *Bacteroides*, *Parabacteroides*, and *Blautia* genera, and eliminate or reduce the populations of *Prevotella*, *Escherichia*, *Clostridium*, and *Sutterella* genera in patients with hyperlipidemia (Li et al., 2016).

Berberine can change the abundance of intestinal mucus produced by *A. muciniphila* bacteria in an animal model for high-fat diets (Zhu et al., 2018). These results indicated that berberine can improve hyperlipidemia by affecting the composition of the intestinal flora. Wang et al. further reported that fecal nitrate reductase (NR) activity in patients with hyperlipidemia was higher than that in healthy individuals. The NR activity of intestinal bacteria plays a key role in promoting the intestinal absorption of berberine. In humans, individuals with high NR activity have been shown to exhibit higher levels of berberine in their blood compared with those with normal fecal NR activity, thereby suggesting a variation in the oral bioavailability of berberine (Wang et al., 2017c).

Other studies have shown that berberine can suppress the production of ATP and NADH levels by bacteria and also the levels of nicotinamide adenine dinucleotide. The ob/ob mice(C57BL/6J) were treated orally with berberine (100 mg/kg/day) for 10 days and their feces sample was taken for the bacterial composition analysis. Of the 50 genera, the abundance of 9 genera increased after berberine treatment. Seven of the nine genera were able to produce butyrate, including *Enterobacter*, *Escherichia − Shigella*, *Incertae sedis*, *Lachnospiraceae FCS020* group, *Akkermansia*, *Clostridium sensu stricto 1*, and *Bacteroides*, with the biggest increase seen in *Enterobacter* and *Escherichia−Shigella* (Wang et al., 2017b). These actions resulted in increased levels of butyryl-CoA, thus promoting the GM to produce butyrate. Once released, butyrate enters the blood and is able to reduce the levels of lipids and glucose. However, the intraperitoneal administration of berberine was unable to increase the levels of butyrate but did reduce the levels of lipids and glucose in the blood. Therefore, berberine appears to act *via* two different models in order to reduce hyperlipidemia: firstly by exerting a direct effect *via* the circulation and secondly, by exerting an indirect effect *via* butyrate produced by GM (Wang et al., 2017b).

## The Effects of Berberine on Liver Disease

Previous research has shown that the liver is exposed to gut-derived bacterial metabolites and products (Eissa et al., 2018). A previous study showed that the concentrations and bioactive metabolites of berberine in the organs were higher than those in the blood during the progression of alcoholic liver disease (ALD) from steatohepatitis to fibrosis, cirrhosis, and then to end-stage liver disease. In addition, berberine was shown to be distributed rapidly in a range of tissues, but predominantly in the liver (Tan et al., 2013).

Berberine has also been shown to significantly reduce inflammation, fibrosis, and the levels of lipid peroxides in the liver (Zhang et al., 2016). The lipid-lowering effect of berberine occurs *via* a series of continuous events, including bile salt hydrolase (BSH) inhibition, significant increases in the levels of tauro-conjugated bile acids (especially TCA), and the activation of the FXR signaling pathway. These events reduce the expression levels of CD36 in the liver which then leads to a reduction in the hepatic uptake of long-chain fatty acids and the modulation of lipid metabolism in the liver (Sun et al., 2017).

Berberine activated a population with immune suppressive function, defined as granulocytic‐ myeloid‐derived suppressor cell (G‐MDSC)‐like population, in the liver of mice with alleviating ALD. Berberine remarkably enhanced the increase of G‐MDSC‐like cells in blood and liver and decreased cytotoxic T cells correspondingly. Moreover, berberine changed the overall gut microbial community, primarily increased the abundance of *A. muciniphila.* Of note, depletion of gut microbiota abolished the inducing effect of berberine on G‐MDSC‐like population, and attenuated its hepatoprotective effect against alcohol in mice, suggesting intestinal flora might be involved in mediating the expansion of this protective population (Li S. et al., 2020). Patients with ALD have been shown to possess an increased abundance of endotoxin-producing *Enterobacteriaceae*, and a reduced abundance of SCFAs-producing bacteria, such as *Lachnospiraceae* and *Ruminococcaceae*. In a previous study, Grander et al. (2018) showed that *A. muciniphila*, a commensal type of bacteria, was associated with intestinal mucous layer in alcoholic hepatitis. These authors showed that clinical stool samples from patients with alcoholic hepatitis had the lowest relative abundance of *A.muciniphila*. Further experiments, using a mouse(C57BL/6J) model of ALD, reported improvements in alcohol-associated hepatic disease and intestinal barrier function following the administration of *A. muciniphila* (Grander et al., 2018). Other studies have shown that berberine can regulate SCFA-producing bacteria (Wang et al., 2017). Human and animal experiments in ALD and cirrhosis have further demonstrated that probiotics, including *Lactobacillaceae* spp. can improve the outcomes of these diseases (Han et al., 2015).

Primary biliary cholangitis (PBC) and primary sclerosing cholangitis (PSC) are both cholestatic liver disorders. Patients with PBC are known to possess reduced populations of beneficial bacteria such as *Acidobacteria*, *Lachnobacterium*, *Bacteroides*, and *Ruminococcus*, and increased populations of pathogens, such as *proteobacteria*, *enterobacteriaceae*, *Veillonella*, *Streptococcus*, and *Klebsiella* (Lv et al., 2016). Berberine is known to exhibit antibacterial activity against *Streptococcus* and *Klebsiella* (Zhou et al., 2016; Du et al., 2020).

The administration of berberine has been shown to restore the relative levels of bifidobacteria, along with the B:F ratio, in mice (BALB/c) that were fed a high-fat diet. These changes subsequently led to an improvement in serum transaminase activity and non-alcoholic fatty liver disease activity scale score (Cao et al., 2016). Furthermore, the expression levels of CD14, IL-1, IL-6, and TNF-α, were significantly downregulated in mice fed on a high-fat diet following berberine treatment (Cao et al., 2016). Berberine can also ameliorate intestinal dysbacteriosis and therefore reduce the liver toxicity caused by pathological/pharmacological intervention. Berberine treatment was also shown to reduce the level of dextran sulfate sodium (DSS)-induced intestinal dysbacteriosis and thus reduce acute liver toxicity (Qin et al., 2018). Therefore, the transplantation of fecal microbiota might represent a useful method to directly explore homeostatic alterations in the GM. Demethyleneberberine (DMB) is an essential *in vivo* metabolite of berberine. DMB has been shown to suppress the activation of hepatic stellate cells (HSCs) and induce apoptosis by regulating the nuclear factor-kappa B (NF-κB) cascade. DMB also has inhibitory effects on collagen synthesis and is able to increase the degradatino of collagen by blocking the transforming growth factor β 1 (TGF-β1)-Smad signaling pathway. In addtion, DMB can reduce the expression of matrix metalloproteinases (MMPs) (Wang et al., 2016).

## The Effects of Berberine on Diabetes Mellitus (DM)

DM is a metabolic disease that is associated with high levels of morbidity and mortality. Alterations and in the GM, along with chronic systemic inflammation, have been shown to lead to DM (Sepp et al., 2014; Cui et al., 2018a). There is increasing evidence to show that changes in the GM are associated with IR and DM. In a previous study, Gao et al. proposed that the “bacteria–mucosal immunity–inflammation–diabetes” axis can be utilized in the prevention and treatment of DM (Gao et al., 2017). Berberine may be efficacious against DM, and has been shown to exert its actions by modulating the GM (Zhang et al., 2015). In a previous study, Cui showed that the relative abundance of *Bacteroidetes* in the rat model of type II diabetes mellitus (T2DM) was lower than in normal rats; this difference was largely abolished following berberine treatment.

Research has shown that berberine fumarate (BF) may play a hypoglycemic role in rats with DM by regulating GM and metabolism. The administration of BF has been shown to significantly ameliorate metabolic disorders, and increase the populations of *Bacteroidetes, Clostridia, Lactobacillales, Prevotellaceae*, and *Alloprevotella*. In addition, the relative abundance of *Clostridia* in the rat intestine was negatively correlated with the host’s blood glucose; BF treatment was shown to reduce the populations of *Bacteroidales, Desulfovibrio*, *Lachnospiraceae*, and *Rikenellaceae* in the rat model of T2DM. Moreover, BF has been shown to reduce inflammation, inhibit the overexpression of toll-like receptor (TLR) and phosphorylated c-Jun N-terminal kinase, and increase the expression of phosphoinositide 3-kinase, glucose transporter-2, and other proteins related to oxidative stress, thus promoting the glucose metabolism (Cui et al., 2018a).

Bacteria in the gut can decompose and metabolize berberine into dihydroberberine, thus preventing the absorption of disaccharides in the intestinal tract (Feng et al., 2015), and increasing the secretion of GLP-1 and GLP-2 to protect pancreatic islet cells and reduce the levels of glucose in the blood (Wei et al., 2014). Furthermore, berberine is able to regulate the expression of a range of related molecules in rats with DM, including the TLR4/MyD88/NF-κB signaling pathway (Gong et al., 2017). In the rat model of T2DM, the bioavailability of berberine is higher than that in normal rats. Furthermore, compared with berberine hydrochloride, berberine organic acid salts (especially BF and berberine succinate) can not only control the levels of blood sugar and avert the occurrence of hyperchloremia, but can also significantly improve the oral bioavailability of berberine (Cui et al., 2018b).

In particular, a number of observational studies have identified an association between elevated circulating levels of branched-chain amino acids (BCAAs) and poor metabolic health. In clinical studies, increased levels of BCAAs in the blood have been positively correlated with insulin resistance (Lynch and Adams, 2014). Berberine has been shown to reduce the relative abundance of BCAA-producing bacteria, including *Clostridiales*; the families of *Streptococcaceae, Clostridiaceae*, the *Streptococcus* genera, and *Prevotella*. Consequently, the increased serum levels of BCAAs induced by the consumption of a high-fat diet are reduced significantly following the administration of berberine. Furthermore, data from both healthy subjects and patients with DM indicate that berberine can improve glycemic control and modulate the circulating levels of BCAAs (Yue et al., 2019). Therefore, it is evident that the hypoglycemic effects of berberine may be related to improvements in the regulation of gut-derived hormones, the weakening of mechanisms in the intestinal mucosal, and the destruction of the immune-barrier.

Both berberine and metformin have been shown to cause changes in more than 20 genera in db/db mice (C57BLKS/JNju, animal models of type 2 diabetes). Both of these treatments caused significant changes in the expression of seven OTUs, including increases in the prevalence of a range of SCFA-producing bacteria, including *Butyricimonas*, *Coprococcus*, and *Ruminococcus* (Zhang et al., 2019). Similar changes were observed in the content of SCFA-producing bacteria in the feces. Both of these treatments led to an increase in the populations of the symbiotic genera *Lactobacillus* and *Akkermansia*. In contrast, both treatments reduced the populations of *Prevotella* and *Proteus*, two types of opportunistic pathogens. Berberine and metformin were able to reduce weight gain and regulate the gut microbiome while suppressing intestinal inflammation and supporting the intestinal barrier (Zhang et al., 2019).

The combination of berberine and stachyose was previously shown to improve glycol-metabolism in T2DM mice (BKS-db) to a better extent than berberine alone (Li et al., 2020); this effect occurred by changes in the regulation of the intestinal microbiota and fecal metabolomics. Following treatment with berberine and stachyose, there was a reduction in the abundance of *Saccharibacteria*, *Deferribacteres*, *Actinobacteria*, and *Firmicutes*, but an increase in the abundance of *Verrucomicrobia*. Furthermore, compared with berberine treatment alone, there was a significant increase in the abundance of *Verrucomicrobia* when stachyose and berberine were administered in combination (Li et al., 2020).

## The Effects of Berberine on Cancer

Colorectal cancer (CRC) is the third most commonly encountered malignant tumor and the fourth leading cause of cancer mortality in the world (Siegel et al., 2019). In 2019, approximately 145,600 new cases of CRC, and 51,020 deaths, were estimated to have involved CRC (Song et al., 2020). An increasing body of data now support the fact that changes in the intestinal microbiome allow environmental risk factors to initiate and promote CRC (Song and Chan, 2019). Previous research has shown that berberine can inhibit the development of colorectal cancer (CRC) (Habtemariam, 2016).

*A. muciniphila* is a gram-negative anaerobic bacterium that is selectively reduced in the fecal microbiota of patients with colitis or colitis-associated cancer (CAC). *amuc_1100* is a special protein that can be isolated from the outer membrane of *A. muciniphila*. Once isolated, *amuc_1100* still exerts biological activity and plays a beneficial role at the temperature used for pasteurization. *A. muciniphila* or *amuc_1100* has been shown to alleviate colitis and CAC, reduce CD8^+^ cytotoxic T lymphocytes (cTls), and the infiltration of macrophages in the colon, and may therefore represent a promising therapeutic target for the treatment of colitis and CRC (Wang et al., 2020). However, research has shown that the population of *Akkermansia* was significantly increased in a mouse model (BALB/c) of CAC fed a high-fat diet (Wu et al., 2016). The Apc^min/+^ mouse model (C57BL/6J) has a tumorigenic phenotype and can develop intestinal tumors; research has shown that high-fat diet could accelerate the process of carcinogenesis. Berberine has been shown to significantly reduce intestinal-tumor development and cause changes in the structure of the GM in Apc^min/+^ mice (C57BL/6J) fed on a high-fat diet (Wang et al., 2018). Berberine can clearly inhibit the increased abundance of *Verrucomicrobia* at the phylum level. At the genus level, berberine can suppress *Akkermansia* and increase the abundance of some SCFA-producing bacteria (Wang et al., 2018).

Berberine has also been shown to promote the interaction between retinoid X receptor alpha (RXRα) and nuclear β-catenin; this leads to the Casitas B-lineage lymphoma (c-Cbl)-mediated degradation of β-catenin, thereby inhibiting the proliferation of colon cancer cells (Ruan et al., 2017).

## The Effects of Berberine on Other Diseases

The modulation of berberine-induced GM plays a significant role in the development of IBD and atherosclerosis (Cui et al., 2018). IBD is caused by dysregulation of the immune responses in the intestinal mucosal in hosts that are genetically susceptible (Strober et al., 2007). Berberine has also been shown to inhibit the production of pro-inflammatory cytokines in colonic macrophages and epithelial cells, and promote apoptosis in the colon macrophages of mice(C57BL/6) treated with DSS. Berberine was also shown to reduce the activation of the signaling pathways that produce proinflammatory cytokines (including mitogen-activated protein kinase and NF-κB) in colonic macrophages and epithelial cells in DSS-treated mice (Yan et al., 2012). In the intestinal mucositis induced by 5-fluorouracil (5-Fu) using rat model, berberine significantly increased the levels of butyrate and glutamine in feces from 5-Fu treated rats. In terms of gut microbiota, berberine enriched the relative abundance of *Firmicutes* and decreased *Proteobacteria* at the phylum level. Meanwhile, berberine increased the proportion of *unclassified_f_ Porphyromonadaceae*, *unclassified_f_ Lachnospiraceae*, *Lactobacillus*, *unclassified_o_Clostridiales*, *Ruminococcus*, *Prevotella*, *Clostridium IV*, and decreased *Escherichia/Shigella* at the genera level (Chen et al., 2020).

Clinical evidence suggests that berberine can reduce endothelial inflammation and improve vascular health (Cicero and Baggioni, 2016). Shi et al. further reported that berberine may modulate the composition of the GM in subjects with atherosclerosis (Shi et al., 2018). Other studies have shown that berberine could be used to treat atherosclerosis by increasing the abundance of *Akkermansia* spp in mice(C57BL) fed a high-fat diet (Zhu et al., 2018). In addition, berberine was shown to reduce HFD-induced metabolic endotoxemia and the expression of proinflammatory cytokines and chemokines in the arteries and in the intestine.

Other research has shown that berberine can reduce the expression of hepatic flavin-containing monooxygenase 3 (FMO3) and the serum levels of proteins involved in the trimethylamine N-oxide FXR signaling pathway (Shi et al., 2018). Similarly, the levels of primary bile acids (e.g., β-muricholic acid and tauroursodeoxycholic acid) were shown to be increased in the livers and sera of mice (C57BL/6) fed berberine; the levels of secondary bile acids (lithocholic acid and T-conjugates) were reduced (Guo et al., 2016). Another study reported that the expression of bile acid-synthetic enzymes (e.g., cytochrome P450 (Cyp)7a1 and Cyp8b1), and an uptake transporter sodium taurocholate co-transporting polypeptide (Ntcp), increased by 39 to 400% in the livers of mice fed high doses of berberine; however, there was no significant change in the expression levels of the nuclear receptor and efflux transporter (Guo et al., 2016).

Berberine treatment has also been shown to increase the abundance of *Akkermansia* in the intestine and alleviate atherosclerosis in Apoe (-/-) mice fed a high-fat diet (Zhu et al., 2018). Collectively, these data indicate that berberine may play different regulatory roles in different disease models and that berberine acts *via* many different systems on a range of targets in the treatment of disease.

## Conclusions

**Figure 1:**
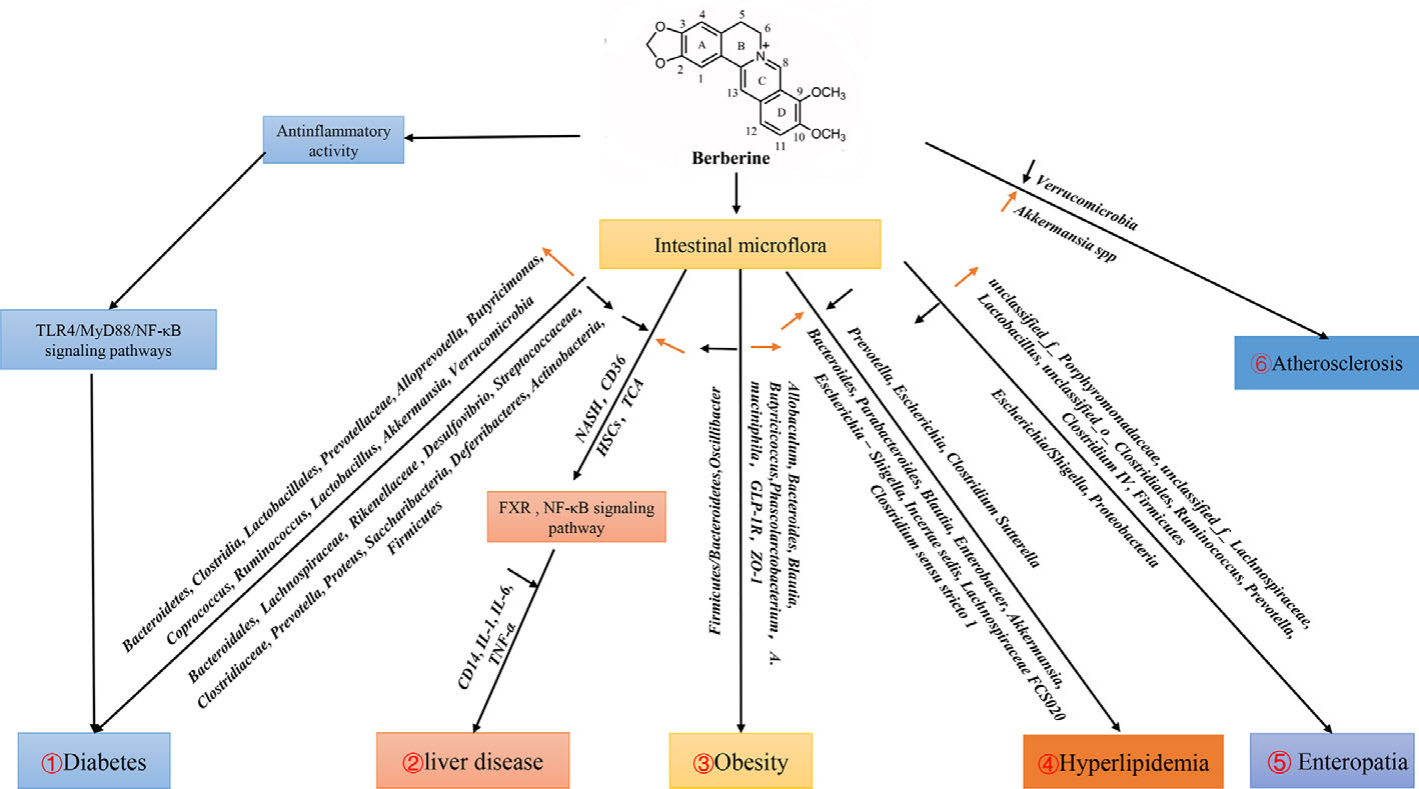
Effects of berberine on diseases by regulating intestinal flora. 1. Berberine can activate TLR4/MyD88/NF-κB signal pathway to play an anti-inflammatory role, and increase the populations of *Bacteroidetes, Clostridia, Lactobacillales, Prevotellaceae, Alloprevotella, Butyricimonas, Coprococcus, Ruminococcus, Lactobacillus, Akkermansia, Verrucomicrobia*, reduce the populations of *Bacteroidales, Lachnospiraceae, Rikenellaceae, Desulfovibrio, Streptococcaceae, Clostridiaceae, Prevotella, Proteus, Saccharibacteria, Deferribacteres, Actinobacteria*, which affect the development of diabetes. 2. Berberine affects liver diseases by regulating FXR and NF-κB signaling pathways through intestinal flora. 3. Berberine down-regulate F:B (*Firmicutes : Bacteroidetes*) and up-regulate SCFA-producing bacteria *Allobaculum, Bacteroides, Blautia, Butyricicoccus, Phascolarctobacterium, A. muciniphila* and GLP-1R in obesity. 4. Berberine regulates hyperlipidemia by reducing *Prevotella, Escherichia, Clostridium Sutterella* and increase *Bacteroides*, *Parabacteroides*, *Blautia*, *Enterobacter*, *Akkermansia*, *Escherichia-Shigella*, *Incertae sedis*, *Lachnospiraceae FCS020, Clostridium sensu stricto 1.* 5. In Enteropatia, berberine enriched the relative abundance of *Firmicutes* and decreased *Proteobacteria* at the phylum level. Meanwhile, berberine increased the propotion of *unclassified_f_Porphyromonadaceae*, *unclassified_f_Lachnospiraceae*, *Lactobacillus*, *unclassified_o_Clostridiales*, *Ruminococcus*, *Prevotella*, *Clostridium IV*, and decreased *Escherichia/Shigella* at the genera level. 6. Berberine affect the development of Atherosclerosis by changing the amount of *Verrucomicrobia*, *Akkermansia* in Apoe (^−/−^) mice fed a high-fat diet in the intestine.

Berberine has various pharmacologic properties and multi-spectrum therapeutic applications (Imenshahidi and Hosseinzadeh, 2019). The GM is an important environmental factor that interacts with its host, and participates in the occurrence and development of various diseases. Several clinical studies have shown that berberine can be used in treatment of different diseases (e.g., DM, hyperlipidemia, cancer, metabolic syndrome, polycystic ovarian syndrome, liver disease) by GM regulation (**Figure 1**).

This review mainly elucidates the role of berberine in a variety of diseases by regulating intestinal flora. However, three main factors need to be addressed. First, there are genetic differences between rodents and humans, so the regulatory effect of the GM through berberine must be demonstrated clinically. Second, better understanding of how the GM regulates glucose metabolism and DM complications is needed. Third, the US Food and Drug Administration has not approved berberine for any indication.

## Author Contributions

This manuscript was designed by LW and XS and drafted by LZ, XW, and RY. FC, YL, ZZ, and ZW contributed to writing. All authors contributed to the article and approved the submitted version.

## Funding

This work was supported by National Natural Science Foundation of China (No. 81902081 and 81871682), China Postdoctoral Science Foundation (No. 2018M640858, 2019T120771), the Natural Science Foundation of Guangdong Province (No. 2020A1515011573 and 2019A1515012068), Pearl River Nova Program of Guangzhou (Grant No.201710010030), Fundamental Research Funds for the Central Universities (No. 19ykpy170, 17ykpy09, No.19ykpy29), National Science and Technology Major Project (No. 2018ZX10101002-001).

## Conflict of Interest

The authors declare that the research was conducted in the absence of any commercial or financial relationships that could be construed as a potential conflict of interest.

## References

[B1] Caliceti C. Franco P. Spinozzi S. Roda A. Cicero A. F. (2016). Berberine: New Insights from Pharmacological Aspects to Clinical Evidences in the Management of Metabolic Disorders. Curr. Med. Chem. 23 (14), 1460–1476. 10.2174/0929867323666160411143314 27063256

[B2] Cao Y. Pan Q. Cai W. Shen F. Chen G. Y. Xu L. M.. (2016). Modulation of Gut Microbiota by Berberine Improves Steatohepatitis in High-Fat Diet-Fed BALB/C Mice. Arch. Iran Med. 19 (3), 197–203. 0161903/AIM.008 26923892

[B3] Chang X. Yan H. Fei J. Jiang M. Zhu H. Lu D.. (2010). Berberine reduces methylation of the MTTP promoter and alleviates fatty liver induced by a high-fat diet in rats. J. Lipid Res. 51 (9), 2504–2515. 10.1194/jlr.M001958 20567026 PMC2918435

[B4] Chen H. Miao H. Feng Y. L. Zhao Y. Y. Lin R. C. (2014). Metabolomics in dyslipidemia. Adv. Clin. Chem. 66, 101–119. 10.1016/b978-0-12-801401-1.00004-9 25344987

[B6] Chen X. Zhang Y. Zhu Z. Liu H. Guo H. Xiong C.. (2016). Protective effect of berberine on doxorubicininduced acute hepatorenal toxicity in rats. Mol. Med. Rep. 13 (5), 3953–3960. 10.3892/mmr.2016.5017 27035423

[B5] Chen H. Zhang F. Li R. Liu Y. Wang X. Zhang X.. (2020). Berberine regulates fecal metabolites to ameliorate 5-fluorouracil induced intestinal mucositis through modulating gut microbiota. Biomed. Pharmacother. = Biome. Pharmacother. 124:109829. 10.1016/j.biopha.2020.109829 31958765

[B7] Cicero A. F. Baggioni A. (2016). Berberine and Its Role in Chronic Disease. Adv. Exp. Med. Biol. 928, 27–45. 10.1007/978-3-319-41334-1_2 27671811

[B8] Cok A. Plaisier C. Salie M. J. Oram D. S. Chenge J. Louters L. L. (2011). Berberine acutely activates the glucose transport activity of GLUT1. Biochimie 93 (7), 1187–1192. 10.1016/j.biochi.2011.04.013 21545824 PMC3526940

[B11] Cui H. Cai Y. Wang L. Jia B. Li J. Zhao S.. (2018). Berberine Regulates Treg/Th17 Balance to Treat Ulcerative Colitis Through Modulating the Gut Microbiota in the Colon. Front. Pharmacol. 9 (1), 103. 10.3389/fphar.2018.00571 29904348 PMC5991375

[B9] Cui H. X. Hu Y. N. Li J. W. Yuan K. (2018a). Hypoglycemic Mechanism of the Berberine Organic Acid Salt under the Synergistic Effect of Intestinal Flora and Oxidative Stress. Oxid. Med. Cell Longev. 2018, 8930374. 10.1155/2018/8930374 30662584 PMC6313974

[B10] Cui H. X. Hu Y. N. Li J. W. Yuan K. Guo Y. (2018b). Preparation and Evaluation of Antidiabetic Agents of Berberine Organic Acid Salts for Enhancing the Bioavailability. Molecules 24 (1), 103. 10.3390/molecules24010103 PMC6337101 30597911

[B12] Derosa G. D’Angelo A. Bonaventura A. Bianchi L. Romano D. Maffioli P. (2013). Effects of berberine on lipid profile in subjects with low cardiovascular risk. Expert Opin. Biol. Ther. 13 (4), 475–482. 10.1517/14712598.2013.776037 23441841

[B13] Ding S. T. Smith E. O. McNeel R. L. Mersmann H. J. (2000). Modulation of porcine adipocyte beta-adrenergic receptors by hormones and butyrate. J. Anim. Sci. 78 (4), 927–933. 10.2527/2000.784927x 10784182

[B14] Du G. F. Le Y. J. Sun X. Yang X. Y. He Q. Y. (2020). Proteomic investigation into the action mechanism of berberine against Streptococcus pyogenes. J. Proteomics 215:103666. 10.1016/j.jprot.2020.103666 31981716

[B15] Eissa L. A. Kenawy H. II El-Karef A. Elsherbiny N. M. El-Mihi K. A. (2018). Antioxidant and anti-inflammatory activities of berberine attenuate hepatic fibrosis induced by thioacetamide injection in rats. Chem. Biol. Interact. 294, 91–100. 10.1016/j.cbi.2018.08.016 30138605

[B16] Everard A. Belzer C. Geurts L. Ouwerkerk J. P. Druart C. Bindels L. B.. (2013). Cross-talk between Akkermansia muciniphila and intestinal epithelium controls diet-induced obesity. Proc. Natl. Acad. Sci. United States America 110 (22), 9066–9071. 10.1073/pnas.1219451110 PMC3670398 23671105

[B17] Feng R. Shou J. W. Zhao Z. X. He C. Y. Ma C. Huang M.. (2015). Transforming berberine into its intestine-absorbable form by the gut microbiota. Sci. Rep. 5:12155. 10.1038/srep12155 26174047 PMC4502414

[B07] Galisteo M. Duarte J. Zarzuelo A. (2008). Effects of dietary fibers on disturbances clustered in the metabolic syndrome. J. Nutr. Biochem. 19 (2), 71–84. 10.1016/j.jnutbio.2007.02.009 17618108

[B18] Gao Z. Li Q. Wu X. Zhao X. Zhao L. Tong X. (2017). New Insights into the Mechanisms of Chinese Herbal Products on Diabetes: A Focus on the “Bacteria-Mucosal Immunity-Inflammation-Diabetes” Axis. J. Immunol. Res. 2017:1813086. 10.1155/2017/1813086 29164155 PMC5661076

[B19] Goldsmith F. Guice J. Page R. Welsh D. A. Taylor C. M. Blanchard E. E.. (2017). Obese ZDF rats fermented resistant starch with effects on gut microbiota but no reduction in abdominal fat. Mol. Nutr. Food Res. 61 (1), 10.1002/mnfr.201501025. 10.1002/mnfr.201501025 PMC5324831 27234399

[B20] Gong J. Hu M. Huang Z. Fang K. Wang D. Chen Q.. (2017). Berberine Attenuates Intestinal Mucosal Barrier Dysfunction in Type 2 Diabetic Rats. Front. Pharmacol. 8:42:42. 10.3389/fphar.2017.00042 28217099 PMC5290458

[B21] Grander C. Adolph T. E. Wieser V. Lowe P. Wrzosek L. Gyongyosi B.. (2018). Recovery of ethanol-induced Akkermansia muciniphila depletion ameliorates alcoholic liver disease. Gut 67 (5), 891–901. 10.1136/gutjnl-2016-313432 28550049

[B22] Guo Y. Zhang Y. Huang W. Selwyn F. P. Klaassen C. D. (2016). Dose-response effect of berberine on bile acid profile and gut microbiota in mice. BMC Complement. Altern. Med. 16 (1), 394. 10.1186/s12906-016-1367-7 27756364 PMC5070223

[B23] Habtemariam S. (2016). Berberine and inflammatory bowel disease: A concise review. Pharmacol. Res. 113 (Pt A), 592–599. 10.1016/j.phrs.2016.09.041 27697643

[B24] Han S. H. Suk K. T. Kim D. J. Kim M. Y. Baik S. K. Kim Y. D.. (2015). Effects of probiotics (cultured Lactobacillus subtilis/Streptococcus faecium) in the treatment of alcoholic hepatitis: randomized-controlled multicenter study. Eur. J. Gastroenterol. Hepatol. 27 (11), 1300–1306. 10.1097/MEG.0000000000000458 26302024

[B25] Hasanein P. Ghafari-Vahed M. Khodadadi I. (2017). Effects of isoquinoline alkaloid berberine on lipid peroxidation, antioxidant defense system, and liver damage induced by lead acetate in rats. Redox Rep. 22 (1), 42–50. 10.1080/13510002.2016.1140406 26871196 PMC6837683

[B26] Imenshahidi M. Hosseinzadeh H. (2019). Berberine and barberry (Berberis vulgaris): A clinical review. Phytother. Res. 33 (3), 504–523. 10.1002/ptr.6252 30637820

[B27] Jin Y. Khadka D. B. Cho W. J. (2016). Pharmacological effects of berberine and its derivatives: a patent update. Expert Opin. Ther. Pat. 26 (2), 229–243. 10.1517/13543776.2016 26610159

[B28] Kamada N. Seo S. U. Chen G. Y. Nunez G. (2013). Role of the gut microbiota in immunity and inflammatory disease. Nat. Rev. Immunol. 13 (5), 321–335. 10.1038/nri3430 23618829

[B29] Kong W. Wei J. Abidi P. Lin M. Inaba S. Li C.. (2004). Berberine is a novel cholesterol-lowering drug working through a unique mechanism distinct from statins. Nat. Med. 10 (12), 1344–1351. 10.1038/nm1135 15531889

[B30] Kuno T. Hirayama-Kurogi M. Ito S. Ohtsuki S. (2016). Effect of Intestinal Flora on Protein Expression of Drug-Metabolizing Enzymes and Transporters in the Liver and Kidney of Germ-Free and Antibiotics-Treated Mice. Mol. Pharm. 13 (8), 2691–2701. 10.1021/acs.molpharmaceut.6b00259 27376980

[B04] Leal-Díaz A. M. Noriega L. G. Torre-Villalvazo I. Torres N. Alemán-Escondrillas G. López-Romero P.. (2016). Aguamiel concentrate from Agave salmiana and its extracted saponins attenuated obesity and hepatic steatosis and increased Akkermansia muciniphila in C57BL6 mice. Sci. Rep. 6, 34242. 10.1038/srep34242 27678062 PMC5039706

[B31] Li C. N. Wang X. Lei L. Liu M. Z. Li R. C. Sun S. J.. (2020). Berberine combined with stachyose induces better glycometabolism than berberine alone through modulating gut microbiota and fecal metabolomics in diabetic mice. Phytotherapy Res. PTR 34 (5), 1166–1174. 10.1002/ptr.6588 PMC7216932 31833107

[B32] Li M. Shu X. Xu H. Zhang C. Yang L. Zhang L.. (2016). Integrative analysis of metabolome and gut microbiota in diet-induced hyperlipidemic rats treated with berberine compounds. J. Transl. Med. 14 (1), 237. 10.1186/s12967-016-0987-5 27495782 PMC4975912

[B33] Li S. Wang N. Tan H. Y. Chueng F. Zhang Z. J. Yuen M. F.. (2020). Modulation of gut microbiota mediates berberine-induced expansion of immuno-suppressive cells to against alcoholic liver disease. Clin. Trans. Med. 10 (4), e112. 10.1002/ctm2.112 PMC7438809 32790968

[B34] Li X. Xu Q. Jiang T. Fang S. Wang G. Zhao J.. (2016). A comparative study of the antidiabetic effects exerted by live and dead multi-strain probiotics in the type 2 diabetes model of mice. Food Funct. 7 (12), 4851–4860. 10.1039/c6fo01147k 27812581

[B35] Louis P. Hold G. L. Flint H. J. (2014). The gut microbiota, bacterial metabolites and colorectal cancer. Nat. Rev. Microbiol. 12 (10), 661–672. 10.1038/nrmicro3344 25198138

[B36] Lv L. X. Fang D. Q. Shi D. Chen D. Y. Yan R. Zhu Y. X.. (2016). Alterations and correlations of the gut microbiome, metabolism and immunity in patients with primary biliary cirrhosis. Environ. Microbiol. 18 (7), 2272–2286. 10.1111/1462-2920.13401 27243236

[B37] Lynch C. J. Adams S. H. (2014). Branched-chain amino acids in metabolic signalling and insulin resistance. Nat. Rev. Endocrinol. 10 (12), 723–736. 10.1038/nrendo.2014 25287287 PMC4424797

[B38] Maslowski K. M. Vieira A. T. Ng A. Kranich J. Sierro F. Yu D.. (2009). Regulation of inflammatory responses by gut microbiota and chemoattractant receptor GPR43. Nature 461 (7268), 1282–1286. 10.1038/nature08530 19865172 PMC3256734

[B39] McNabney S. M. Henagan T. M. (2017). Short Chain Fatty Acids in the Colon and Peripheral Tissues: A Focus on Butyrate, Colon Cancer, Obesity and Insulin Resistance. Nutrients 9 (12), 1348. 10.3390/nu9121348 PMC5748798 29231905

[B05] Mollica M. P. Mattace Raso G. Cavaliere G. Trinchese G. De Filippo C. Aceto S.. (2017). Butyrate regulates liver mitochondrial function, efficiency, and dynamics in insulin-resistant obese mice. Diabetes 66 (5), 1405–1418. 10.2337/db16-0924 28223285

[B08] Nilsson A. C. Östman E. M. Knudsen K. E. Holst J. J. Björck I. M. (2010). A cereal-based evening meal rich in indigestible carbohydrates increases plasma butyrate the next morning. J. Nutr. 140 (11), 1932–1936. 10.3945/jn.110.123604 20810606

[B40] Qin C. Zhang H. Zhao L. Zeng M. Huang W. Fu G.. (2018). Microbiota transplantation reveals beneficial impact of berberine on hepatotoxicity by improving gut homeostasis. Sci. China Life Sci. 61 (12), 1537–1544. 10.1007/s11427-017-9202-0 29270794

[B02] Roediger W. E. (1982). Utilization of nutrients by isolated epithelial cells of the rat colon. Gastroenterology 83 (2), 424–429. 7084619

[B41] Roudini L. NayebZadeh Eidgahi N. Rahimi H. R. Saberi M. R. Amiri Tehranizadeh Z. Beigoli S.. (2019). Determining the interaction behavior of calf thymus DNA with berberine hydrochloride in the presence of linker histone: a biophysical study. J. Biomol. Struct. Dyn. 38 (2), 364–381. 10.1080/07391102.2019 30773095

[B42] Round J. L. Mazmanian S. K. (2009). The gut microbiota shapes intestinal immune responses during health and disease. Nat. Rev. Immunol. 9 (5), 313–323. 10.1038/nri2515 19343057 PMC4095778

[B43] Ruan H. Zhan Y. Y. Hou J. Xu B. Chen B. Tian Y.. (2017). Berberine binds RXRalpha to suppress beta-catenin signaling in colon cancer cells. Oncogene 36 (50), 6906–6918. 10.1038/onc.2017.296 28846104 PMC5735301

[B03] Schneeberger M. Everard A. Gómez-Valadés A. G. Matamoros S. Ramírez S. Delzenne N. M.. (2015). Akkermansia muciniphila inversely correlates with the onset of inflammation, altered adipose tissue metabolism and metabolic disorders during obesity in mice. Sci. Rep. 5, 16643. 10.1038/srep16643 26563823 PMC4643218

[B44] Sekirov I. Russell S. L. Antunes L. C. Finlay B. B. (2010). Gut microbiota in health and disease. Physiol. Rev. 90 (3), 859–904. 10.1152/physrev.00045.2009 20664075

[B45] Sepp E. Kolk H. Loivukene K. Mikelsaar M. (2014). Higher blood glucose level associated with body mass index and gut microbiota in elderly people. Microb. Ecol. Health Dis. 25, 10.3402/mehd.v25.22857. 10.3402/mehd.v25.22857 PMC4048595 24936169

[B46] Shen J. Tong X. Sud N. Khound R. Song Y. Maldonado-Gomez M. X.. (2016). Low-Density Lipoprotein Receptor Signaling Mediates the Triglyceride-Lowering Action of Akkermansia muciniphila in Genetic-Induced Hyperlipidemia. Arterioscler. Thromb. Vasc. Biol. 36 (7), 1448–1456. 10.1161/ATVBAHA.116.307597 27230129

[B47] Shi Y. Hu J. Geng J. Hu T. Wang B. Yan W.. (2018). Berberine treatment reduces atherosclerosis by mediating gut microbiota in Aope-/- mice. BioMed. Pharmacother. 107, 1556–1563. 10.1016/j.biopha.2018.08.148 30257374

[B48] Siegel R. L. Miller K. D. Jemal A. (2019). Cancer statistics.(2019). CA Cancer J. Clin. 69 (1), 7–34. 10.3322/caac.21551 30620402

[B49] Song M. Chan A. T. (2019). Environmental Factors, Gut Microbiota, and Colorectal Cancer Prevention. Clin. Gastroenterol. Hepatol. Off. Clin. Pract. J. Am. Gastroenterol. Assoc. 17 (2), 275–289. 10.1016/j.cgh.2018.07.012 PMC6314893 30031175

[B50] Song M. Chan A. T. Sun J. (2020). Influence of the Gut Microbiome, Diet, and Environment on Risk of Colorectal Cancer. Gastroenterology 158 (2), 322–340. 10.1053/j.gastro.2019.06.048 31586566 PMC6957737

[B51] Strober W. Fuss I. Mannon P. (2007). The fundamental basis of inflammatory bowel disease. J. Clin. Invest. 117 (3), 514–521. 10.1172/JCI30587 17332878 PMC1804356

[B52] Sun H. Wang N. Cang Z. Zhu C. Zhao L. Nie X.. (2016). Modulation of Microbiota-Gut-Brain Axis by Berberine Resulting in Improved Metabolic Status in High-Fat Diet-Fed Rats. Obes. Facts 9 (6), 365–378. 10.1159/000449507 27898425 PMC5644798

[B53] Sun R. Yang N. Kong B. Cao B. Feng D. Yu X.. (2017). Orally Administered Berberine Modulates Hepatic Lipid Metabolism by Altering Microbial Bile Acid Metabolism and the Intestinal FXR Signaling Pathway. Mol. Pharmacol. 91 (2), 110–122. 10.1124/mol.116.106617 27932556 PMC5267522

[B54] Tan X. S. Ma J. Y. Feng R. Ma C. Chen W. J. Sun Y. P.. (2013). Tissue distribution of berberine and its metabolites after oral administration in rats. PloS One 8 (10), e77969. 10.1371/journal.pone.0077969 24205048 PMC3815028

[B55] Tian Y. Cai J. Gui W. Nichols R. G. Koo I. Zhang J.. (2019). Berberine Directly Affects the Gut Microbiota to Promote Intestinal Farnesoid X Receptor Activation. Drug Metab. Dispos. 47 (2), 86–93. 10.1124/dmd.118.083691 30409838 PMC6323626

[B56] Turnbaugh P. J. Ley R. E. Mahowald M. A. Magrini V. Mardis E. R. Gordon J. II (2006). An obesity-associated gut microbiome with increased capacity for energy harvest. Nature 444 (7122), 1027–1031. 10.1038/nature05414 17183312

[B57] Vidrine K. Ye J. Martin R. J. McCutcheon K. L. Raggio A. M. Pelkman C.. (2014). Resistant starch from high amylose maize (HAM-RS2) and dietary butyrate reduce abdominal fat by a different apparent mechanism. Obes. (Silver Spring Md.) 22 (2), 344–348. 10.1002/oby.20501 23630079

[B58] Walker A. W. Parkhill J. (2013). Microbiology. Fighting Obes. Bacteria Sci. 341 (6150), 1069–1070. 10.1126/science.1243787 24009379

[B64] Wang Y. Jia X. Ghanam K. Beaurepaire C. Zidichouski J. Miller L. (2010). Berberine and plant stanols synergistically inhibit cholesterol absorption in hamsters. Atherosclerosis 209 (1), 111–117. 10.1016/j.atherosclerosis.2009.08.050 19782362

[B63] Wang X. He G. Peng Y. Zhong W. Wang Y. Zhang B. (2015). Sodium butyrate alleviates adipocyte inflammation by inhibiting NLRP3 pathway. Sci. Rep. 5:12676. 10.1038/srep12676 26234821 PMC4522654

[B67] Wang Y. Zhao Z. Yan Y. Qiang X. Zhou C. Li R.. (2016). Demethyleneberberine Protects against Hepatic Fibrosis in Mice by Modulating NF-kappaB Signaling. Int. J. Mol. Sci. 17 (7), 1036. 10.3390/ijms17071036 PMC4964412 27376272

[B62] Wang L. L. Guo H. H. Huang S. Feng C. L. Han Y. X. Jiang J. D. (2017). Comprehensive evaluation of SCFA production in the intestinal bacteria regulated by berberine using gas-chromatography combined with polymerase chain reaction. J. Chromatogr. B Anal. Technol. Biomed. Life Sci. 1057, 70–80 1057:70–80. 10.1016/j.jchromb.2017.05.004 Sci. 2017 Jul 1. 28505492

[B60] Wang K. Feng X. Chai L. Cao S. Qiu F. (2017a). The metabolism of berberine and its contribution to the pharmacological effects. Drug Metab. Rev. 49 (2), 139–157. 10.1080/03602532.2017.1306544 28290706

[B65] Wang Y. Shou J. W. Li X. Y. Zhao Z. X. Fu J. He C. Y.. (2017b). Berberine-induced bioactive metabolites of the gut microbiota improve energy metabolism. Metabolism 70, 72–84. 10.1016/j.metabol.2017.02.003 28403947

[B66] Wang Y. Tong Q. Shou J. W. Zhao Z. X. Li X. Y. Zhang X. F.. (2017c). Gut Microbiota-Mediated Personalized Treatment of Hyperlipidemia Using Berberine. Theranostics 7 (9), 2443–2451. 10.7150/thno.18290 28744326 PMC5525748

[B59] Wang H. Guan L. Li J. Lai M. Wen X. (2018). The Effects of Berberine on the Gut Microbiota in Apc (min/+) Mice Fed with a High Fat Diet. Molecules 23 (9), 2298. 10.3390/molecules23092298 PMC6225274 30205580

[B61] Wang L. Tang L. Feng Y. Zhao S. Han M. Zhang C.. (2020). A purified membrane protein from akkermansia muciniphila or the pasteurised bacterium blunts colitis associated tumourigenesis by modulation of cd8+ t cells in mice. Gut 69 (11), 1988–1997. 10.1136/gutjnl-2019-320105 32169907 PMC7569398

[B68] Wei S. C. Dong S. Xu L. J. Zhang C. Y. (2014). Intestinal absorption of berberine and 8-hydroxy dihydroberberine and their effects on sugar absorption in rat small intestine. J. Huazhong Univ. Sci. Technolog. Med. Sci. 34 (2), 186–189. 10.1007/s11596-014-1256-6 24710930

[B69] Wu M. Wu Y. Deng B. Li J. Zhong G. (2016). Isoliquiritigenin decreases the incidence of colitis-associated colorectal cancer by modulating the intestinal microbiota. Oncotarget 7 (51), 85318. 10.18632/oncotarget.13347 27863401 PMC5356739

[B70] Yan F. Wang L. Shi Y. Cao H. Liu L. Washington M. K.. (2012). Berberine promotes recovery of colitis and inhibits inflammatory responses in colonic macrophages and epithelial cells in DSS-treated mice. Am. J. Physiol. Gastrointest. Liver Physiol. 302 (5), G504–G514. 10.1152/ajpgi.00312.2011 22173918 PMC3311435

[B71] Yang Y. Kang N. Xia H. Li J. Chen L. Qiu F. (2010). Metabolites of protoberberine alkaloids in human urine following oral administration of Coptidis Rhizoma decoction. Planta. Med. 76 (16), 1859–1863. 10.1055/s-0030-1250053 20549593

[B72] Yao J. Kong W. Jiang J. (2015). Learning from berberine: Treating chronic diseases through multiple targets. Sci. China Life Sci. 58 (9), 854–859. 10.1007/s11427-013-4568-z 24174332

[B73] Yue S. J. Liu J. Wang A. T. Meng X. T. Yang Z. R. Peng C.. (2019). Berberine alleviates insulin resistance by reducing peripheral branched-chain am ino acids. Am. J. Physiol. Endocrinol. Metab. 2019 Jan 1;316 (1), E73–E85. 10.1152/ajpendo.00256.2018 30422704

[B77] Zhang X. Zhao Y. Zhang M. Pang X. Xu J. Kang C.. (2012). Structural changes of gut microbiota during berberine-mediated prevention of obesity and insulin resistance in high-fat diet-fed rats. PloS One 7 (8), e42529. 10.1371/journal.pone.0042529 22880019 PMC3411811

[B76] Zhang X. Zhao Y. Xu J. Xue Z. Zhang M. Pang X.. (2015). Modulation of gut microbiota by berberine and metformin during the treatment of high-fat diet-induced obesity in rats. Sci. Rep. 5:14405. 10.1038/srep14405 26396057 PMC4585776

[B78] Zhang Z. Li B. Meng X. Yao S. Jin L. Yang J.. (2016). Berberine prevents progression from hepatic steatosis to steatohepatitis and fibrosis by reducing endoplasmic reticulum stress. Sci. Rep. 6:20848. 10.1038/srep20848 26857750 PMC4746620

[B74] Zhang L. Du J. Yano N. Wang H. Zhao Y. T. Dubielecka P. M.. (2017). Sodium Butyrate Protects -Against High Fat Diet-Induced Cardiac Dysfunction and Metabolic Disorders in Type II Diabetic Mice. J. Cell. Biochem. 118 (8), 2395–2408. 10.1002/jcb.25902 28109123 PMC5462877

[B75] Zhang W. Xu J. H. Yu T. Chen Q. K. (2019). Effects of berberine and metformin on intestinal inflammation and gut microbiome composition in db/db mice. Biomed. Pharmacother. = Biome. Pharmacother. 118:109131. 10.1016/j.biopha.2019.109131 31545226

[B79] Zhou X. Y. Ye X. G. He L. T. Zhang S. R. Wang R. L. Zhou J.. (2016). In vitro characterization and inhibition of the interaction between ciprofloxacin and berberine against multidrug-resistant Klebsiella pneumoniae. J. Antibiot. 69 (10), 741–746. 10.1038/ja.2016.15 PMC5399161 26932407

[B80] Zhu L. Zhang D. Zhu H. Zhu J. Weng S. Dong L.. (2018). Berberine treatment increases Akkermansia in the gut and improves high-fat diet-induced atherosclerosis in Apoe-/- mice. Atherosclerosis 268, 117–126. 10.1016/j.atherosclerosis.2017.11.023 29202334

